# The impact of SARS-COV-2 infection on menstruation

**DOI:** 10.1186/s12905-023-02697-2

**Published:** 2023-11-16

**Authors:** Xiaozhu Zhong, Keji Lu, Weiying Liang, Luozi Jihu, Anqi Zeng, Miao Ding, Dongmei Chen, Meiqing Xie

**Affiliations:** 1 Department of Obstetrics and Gynecology, Sun Yat-sen Memorial Hospital, Sun Yat-sen University, Guangzhou City, 510120 China; 2 Department of Obstetrics & Gynaecology, The First Affiliated Hospital of Sun Yat-sen University, Guangzhou City, 510080 China

**Keywords:** COVID-19, Abnormal uterine bleeding, Menstrual cycle

## Abstract

**Background:**

Recent study has demonstrated that the GnRH system in patients with post-COVID syndrome may be influenced by SARS-CoV-2. However, the impact of COVID-19 infection on women’s menstruation is still unknown.

**Objective:**

We aimed to investigate the the relationship between coronavirus disease 2019 (COVID-19) and menstruation in premenopausal women.

**Methods:**

This was a retrospective cohort study. Pre-menopausal women were invited to participate in the online questionnaire on wechat. Participants were divided into four groups according to whether they were infected with severe acute respiratory syndrome coronavirus-2 (SARS-COV-2) and whether they had menstrual changes during the pandemic. Sociodemographic characteristics, history of COVID-19, menstruation and menstrual changes of the participants were collected. Statistical analyses were performed using SPSS, version 25.0 (SPSS Inc., Chicago, IL, USA).

**Results:**

A total of 1946 women were included in the study. 1800 participants had been or were currently infected with SARS-COV-2, and 146 people had not been infected. Among 1800 patients with COVID-19, 666 (37.0%) had changes in menstruation, and 1134 (63.0%) did not, which was significantly higher than the uninfected participants (c^2^ = 12.161, P = 0.000). The proportion of participants with menstrual cycle changes (450/67.6%) is larger than that of uninfected participants (c^2^ = 6.904, P = 0.009). COVID-19 vaccination was associated with lower odds of menstrual cycle change (OR, 0.855; 95% CI, 0.750–0.976). Participants who reported chest pain (OR, 1.750, 95% CI, 1.209–2.533) and dyspnea (OR, 1.446; 95% CI, 1.052–1.988) during infection had greater odds of changes to their menstrual cycle compared with the participants who did not.

**Conclusions:**

The association between the COVID-19 and increased prevalence of menstrual cycle irregularity. COVID-19 vaccination is a protective factor in the long term, and participants with chest pain and dyspnea are more likely to develop AUB.

## Background

With the spread of severe acute respiratory syndrome coronavirus 2 (SARS-CoV-2) around the world, many women have been affected by this virus. SARS-CoV-2 is a single-stranded RNA virus that is part of the Corona family. It is thought to initially cause respiratory illness, followed by various symptoms such as fever, chills, and loss of smell or taste [1]. More than 200 symptoms have been identified in multiple organ systems after SARS-CoV-2 infection, including those in the reproductive system [2, 3]. Menstruation is a complex process involving hormonal changes in the body that can affect mood, energy levels and physical health. Standardized parameters for typical menstruation have been defined by the International Federation of Gynecology and Obstetrics (FIGO) regarding menstrual frequency, duration, regularity and blood volume, and deviation from these may constitute abnormal uterine bleeding (AUB), which carries a financial and quality-of-life burden to women [4, 5].

Various studies have shown that COVID-19 vaccination and SARS-CoV-2 infection temporarily altered menstrual cycles [6–8], but there was not much research on COVID-19 infection and changes in menstruation. Moreover, many studies lack an uninfected control group to identify COVID-19-associated cycle changes. We observed that most women experienced menstrual changes after COVID-19 infection. Therefore, we conducted a cohort study to investigate the relationship between COVID-19 and menstruation in premenopausal women.

## Methods

### Study design, population and data collection

This was a retrospective cohort study. Sample size calculation was performed using PASS version 15.0. When calculating the sample size, inspection level α = 0.05 and power of test 1-β = 0.90 were set. The study was approved by the institutional review board of Sun Yat-Sen Memorial Hospital, Sun Yat-Sen University, Guangzhou, Guangdong, China (Ethical approvement: SYSKY-2023-092-01.

Pre-menopausal women with or without a history of COVID-19 were invited to participate in the online questionnaire on wechat from January to February 2023. Except for logical errors in filling out the questionnaire, we subsequently excluded participants who: (1) were pregnant, lactating or perimenopausal, (2) were taking oral contraceptives, (3) experienced hysterectomy or oophorectomy, (4) had AUB prior to infection. 2259 participants had completed the online questionnaire, the response rate was 99.7% (2259/2265). Participants were divided into four groups according to whether they were infected with SARS-CoV-2 and whether they had menstrual changes during the pandemic. The exposure variable was SARS-CoV-2 infection. Group 1 was COVID-19 with AUB. Group 2 was COVID-19 without AUB. Group 3 was non-COVID-19 combined with AUB. Group 4 was non-COVID-19 without AUB.

The questionnaires included age, region, occupation, comorbidities, daily sleep time, history of COVID-19 (including symptoms of infection, severity of COVID-19, medication during infection), time between infection and last menstrual period, menstruation (including menstrual cycle, period, blood volume, dysmenorrhea) and menstrual changes. In order to help participants recall their menstruation, we set specific options in the questionnaire, such as the use of tampon or pad per day, the size of blood clots, the severity of dysmenorrhea, etc. The primary outcome was the incidence of AUB. The secondary outcomes included age, comorbidities, COVID-19 vaccination, severity of COVID-19 illness, menstrual characteristics, etc.

### Statistical analysis

Categorical variables were transformed into counts and percentages (%), and were compared by chi-squared test or rank sum test. Logistic regression analysis was used to determine the predictors of AUB after SARS-CoV-2 infection. Statistical analyses were performed using SPSS, version 25.0 (SPSS Inc., Chicago, IL, USA). *P* < 0.05 was considered statistically significant.

## Results

### Basic information of the participants

**Fig. 1:**
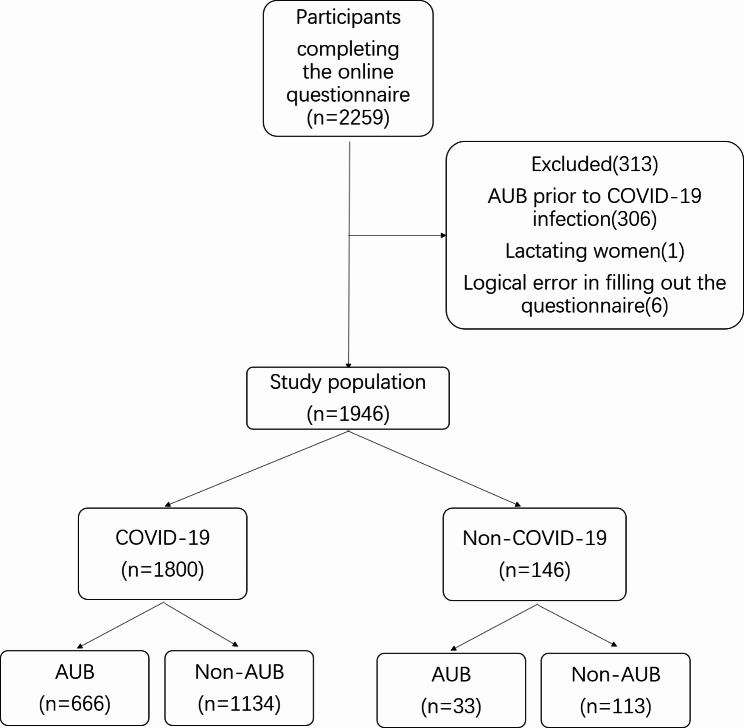
Flowchart of participants included in the study

A total of 1946 women were included in the study. 1800 participants were infected with SARS-COV-2 and 146 were not. AUB occurred in 666 women in the COVID-19 group and 33 women in the non-COVID-19 group (Fig. 1). Sociodemographic and health characteristics of the participants were shown in Table 1. 45.5% of the participants were 26–35 years old, sleeping time of 75.1% participants were 6.1-8 h per day, and 83.7% were free of comorbidities. 69.2% of the participants had received three doses of the inactivated SARS-CoV-2 vaccine and it had been more than 9 months since their last vaccination. In terms of occupation, health care workers (801/41.2%) accounted for the largest proportion of participants. Most of the participants are from southern China (1373/70.6%). Menstrual characteristics of the participants were shown in Table 2. Among the 1964 women who participated in the study, 1744/88.8% had a menstrual cycle of 21–35 days, 1449/73.8% had approximately 5–7 days of menstrual period, 1142/58.1% had moderate menstrual blood volume, and 1265/64.4% showed no signs of dysmenorrhea.

**Table 1:** Sociodemographic and health characteristics of the participants

Characteristics, n(%)	COVID-19			Non-COVID-19	
	AUB(n = 666)	Non-AUB(n = 1134)		AUB(n = 33)	Non-AUB(n = 113)
**Age(years)**					
<20	5 (0.7)	20 (1.7)		3 (9.0)	3 (2.6)
20–25	95 (14.2)	127 (11.1)		8 (24.2)	15 (13.2)
26–30	179 (26.8)	253 (22.3)		6 (18.1)	26 (23.0)
31–35	145 (21.7)	248 (21.8)		7 (21.2)	22 (19.4)
36–40	114 (17.1)	220 (19.4)		2 (6.0)	18 (15.9)
41–45	74 (11.1)	182 (16.0)		2 (6.0)	14 (12.3)
>45	54 (8.1)	84 (7.4)		5 (15.1)	15 (13.2)
**Daily sleep time(hours)**					
<5	10 (1.5)	16 (1.4)		0	0
5.1-6	66 (9.9)	143 (12.6)		4 (12.1)	11 (9.7)
6.1-7	240 36.0)	397 (35.0)		14 (42.4)	45 (39.8)
7.1-8	269 (40.3)	440 (38.8)		12 (36.3)	45 (39.8)
>8	81 (12.1)	138 (12.1)		3 (9.0)	12 (10.6)
**Comorbidities**					
None	547 (82.1)	960 (84.7)		27 (81.8)	94 (83.2)
Hypertension	10 (1.5)	20 (1.8)		0	4 (3.5)
Diabetes	6 (0.9)	9 (0.8)		0	0
Cardiovascular disease	5 (0.8)	16 (1.4)		0	0
Pulmonary disease	15 (2.3)	12 (1.1)		2 (6.0)	0
Chronic kidney disease	1 (0.2)	3 (0.3)		0	0
Chronic liver disease	26 (3.9)	27 (2.4)		2 (6.0)	4 (3.5)
Autoimmune diseases	21 (3.2)	29 (2.6)		1 (3.0)	9 (8.0)
Carcinoma	10 (1.5)	22 (1.9)		1 (3.0)	1 (0.9)
others	50 (7.5)	66 (5.8)		0	4 (3.5)
**COVID-19 vaccination**					
One dose	15 (2.3)	18 (1.6)		1 (3.0)	1 (0.9)
Two doses	132 (19.8)	177 (15.6)		7 (21.2)	21 (18.6)
Three doses	447 (67.1)	818 (72.1)		16 (48.5)	66 (58.4)
Four doses	36 (5.4)	75 (6.6)		7 (21.2)	20 (17.7)
Not vaccinated	36 (5.4)	46 (4.1)		2 (6.0)	5 (4.4)
**Time since the last vaccination**					
0–3 months	61 (9.2)	116 (10.2)		8 (24.2)	22 (19.5)
3–6 months	33 (5.0)	49 (4.32)		1 (3.0)	5 (4.4)
6–9 months	87 (13.1)	148 (13.1)		7 (21.2)	11 (9.7)
>9 months	449 (67.4)	774 (68.3)		15 (15.5)	70 (61.9)

**Table 2:** Menstrual characteristics of the participants

Menstruation characteristics n(%)	COVID-19			Non-COVID-19	
	AUB(n = 666)	Non-AUB(n = 1134)		AUB(n = 33)	Non-AUB(n = 113)
**Menstrual cycle**					
<21 days	13 (2.0)	36 (3.2)	1 (3.0)		4 (3.5)
21–27 days	211 (31.7)	397 (35.0)	8 (24.2)		46 (40.7)
28–35 days	387 (58.1)	627 (55.3)	14 (42.4)		54 (47.8)
36 days-2 months	37 (5.6)	54 (4.8)	9 (27.3)		7 (6.2)
2–3 months	4 (0.6)	6 (0.5)	0		1 (0.9)
> 3 months	14 (2.1)	14 (1.2)	1 (3.0)		1 (0.9)
**Menstrual period**					
2–4 days	119 (17.9)	185 (16.3)	4 (12.1)		25 (22.1)
5–7 days	494 (74.2)	852 (75.1)	26 (78.8)		77 (68.1)
8–10 days	46 (6.9)	88 (7.8)	2 (6.0)		11 (9.7)
> 10 days	7 (1.1)	9 (0.8)	1 (3.0)		0
**Menstrual volume**					
Very little	11 (1.7)	21 (1.9)	1 (3.0)		1 (0.9)
Less than normal	164 (24.6)	240 (21.2)	7 (21.2)		25 (22.1)
Normal	361 (54.2)	693 (61.1)	17 (51.5)		71 (62.8)
More than normal	107 (16.1)	160 (14.1)	7 (21.2)		15 (13.3)
Heavy	23 (3.5)	20 (1.8)	1 (3.0)		1 (0.9)
**Dysmenorrhea**					
Yes	266 (39.9)	362 (31.9)	10 (30.3)		43 (38.1)
no	400 (60.1)	772 (68.1)	23 (69.7)		70 (61.9)

### Infection with SARS-CoV-2 showed increased incidence of AUB

Table 3 showed the comparison of menstrual changes between COVID-19 and non-COVID-19 group. Participants infected with SARS-COV-2 reported a larger proportion of AUB (37.0% vs. 22.6%, χ^2^ = 12.161, P = 0.000) compared to those without COVID-19, of which only menstrual cycle was significantly different (67.6% vs. 45.5%, χ^2^ = 6.904, P = 0.009). Among the 666 participants in COVID-19 group, 191/28.7% had a shorter cycle, 194/29.1% had a longer cycle while 65/9.8% reported not menstruating after infection. In the non-COVID-19 group, 15/45.5% women reported menstrual cycle changes that 3/9.1% women had a shorter cycle and 12/36.4% women had a longer one. There was no difference in menstrual period, menstrual blood volume, intermenstrual bleeding and dysmenorrhea between the COVID-19 group and the non-COVID group. There was no difference in the timing of being infected during the menstrual cycle between women with AUB and without AUB after SARS-COV-2 infection [follicular phase (41.0% vs. 39.8%), ovulation phase (10.1% vs. 9.0%), luteal phase (48.9% vs. 51.2%), χ^2^ = 1.098, *P* = 0.578].

**Table 3:** Comparison of abnormal uterine bleeding in participants

Menstrual characteristics n(%)	COVID-19(n = 1800)				Non-COVID-19 (n = 146)		c2	*P* value
	AUB		Non-AUB		AUB	Non-AUB		
	666(37.0)		1134(63.0)	33(22.6)		113(77.4)	12.161	0.000
	Abnormal	normal		Abnormal	normal			
Menstrual cycle	450(67.6)	216(32.4)		15(45.5)	18(54.5)		6.904	0.009
Menstrual period	290(43.5)	376(56.5)		10(30.3)	23(69.7)		2.250	0.134
Menstrual volume	462(69.4)	204(30.6)		19(57.6)	14(42.4)		2.038	0.153
Intermenstrual bleeding	226(33.9)	440(66.1)		12(36.4)	21(63.6)		0.083	0.774
Dysmenorrhea	170(25.5)	496(74.5)		10(30.3)	23(69.7)		0.375	0.540

### Analysis of risk factors of menstrual cycle changes

Symptoms and treatment of COVID-19 infection were shown in Table 4. Among the 1800 COVID-19 patients in the study, fever, myalgia or fatigue, coughs, headaches, sore throat and runny nose were common symptoms. The illness severity of participants was mostly mild (644/96.7%). The infected are routinely treated with non-steroidal anti-inflammatory drugs (NSAIDS) and Chinese patent medicines.

**Table 4:** Comparison of characteristics of SARS-CoV-2 infection between AUB and non-AUB participants

Characteristics n(%)	COVID-19		c2	*P* value
	AUB(n = 666)	Non-AUB(n = 1134)		
**Signs and symptoms**				
Asymptomatic	4(0.6)	16(1.4)	2.507	0.113
Fever	589 (88.4)	937 (82.6)	10.977	0.001
Headache	453 (68.0)	706 (62.3)	6.072	0.014
Myalgia or fatigue	399 (59.9)	592 (52.2)	10.067	0.002
Nasal congestion and runny nose	425(63.8)	669(59.0)	4.088	0.043
Sore throat	429 (64.4)	699 (61.6)	1.380	0.240
Cough	545 (81.8)	888 (78.3)	3.212	0.073
Chest pain	79(11.9)	65(5.7)	21.421	0.000
Loss of sense of smell and taste	280 (42.0)	430 (37.9)	2.986	0.084
Dyspnea	105 (15.8)	65 (5.7)	18.335	0.000
Diarrhea	127 (19.1)	154 (13.6)	9.595	0.002
**Illness Severity**				
Asymptomatic	9 (1.4)	21 (1.9)		
Mild	644 (96.7)	1101 (97.1)	2.666^a^	0.118
Severe	13 (2.0)	12 (1.1)		
**Medications**				
Non-steroid anti-inflammatory drug	444 (66.7)	840 (74.1)	11.258	0.001
Anti-cough medicine	198 (29.7)	308(27.2)	1.371	0.242
Chinese patent medicine	331 (50.0)	568 (50.1)	0.025	0.874
Chinese herbal medicine	98 (14.7)	164 (14.5)	0.022	0.883

^*a*^ *H value analyzed by rank sum test*

In the logistic regression analysis, COVID-19 vaccination was associated with lower odds of experiencing AUB (OR, 0.855; 95% CI, 0.750–0.976). Participants who reported chest pain (OR, 1.750, 95% CI, 1.209–2.533) and dyspnea (OR, 1.446; 95% CI, 1.052–1.988) during infection had greater odds of AUB compared with those who did not (Table 5).

**Table 5:** Logistic regression analysis applied to determine the predictors of AUB after SARS-CoV-2 infection

Predictor	OR	Odds ratio (95% CI)	*P* value
Age	0.941	0.877–1.009	0.088
Daily sleep time	1.052	0.941–1.175	0.374
No comorbidities	0.980	0.513–1.875	0.952
Hypertension	1.074	0.397–2.903	0.888
Diabetes	1.165	0.368–3.692	0.795
Cardiovascular disease	0.508	0.157–1.649	0.260
Pulmonary disease	1.961	0.776–4.958	0.155
Chronic kidney disease	0.472	0.045–4.977	0.532
Chronic liver Disease	1.640	0.786–3.425	0.188
Autoimmune diseases	1.124	0.523–2.417	0.764
Carcinoma	0.747	0.284–1.965	0.555
COVID-19 vaccination	0.855	0.75–0.976	0.020
Time since the last vaccination	1.000	0.916–1.091	0.998
COVID-19 illness Severity	0.796	0.436–1.453	0.457
Fever	1.356	0.992–1.854	0.056
Headache	1.019	0.815–1.273	0.869
Myalgia or fatigue	1.175	0.947–1.457	0.143
Nasal congestion and runny nose	1.028	0.831–1.272	0.796
Diarrhoea	1.237	0.937–1.633	0.133
Dyspnea	1.446	1.052–1.988	0.023
Chest pain	1.750	1.209–2.533	0.003
Loss of sense of smell and taste	1.005	0.815–1.239	0.965
Non-steroid anti-inflammatory drug	0.671	0.430–1.049	0.080

## Discussion

The COVID-19 pandemic is associated with increased menstrual disorders [9, 10]. The menstrual cycle is regulated by a complex interaction of hormones that linked to the hypothalamic-pituitary-ovarian (HPO) axis, immune, vascular and coagulation systems. Given the heterogeneity of menstrual cycles, a particular challenge was to determine how much COVID-19 could contribute to menstrual changes rather than background variation [10]. COVID-19 vaccination, COVID-19 illness, COVID-19 therapy and stress during the COVID-19 pandemic must be considered when investigating the impact of SARS-CoV-2 infection on the menstruation.

The menstrual cycle of women with high levels of stress will be seriously disturbed. Different types and levels of stress factors have different effects on menstrual frequency, menstrual bleeding and menstrual period [11]. It has been proved that the aggravation of premenstrual syndrome and dysmenorrhea is related to stress, psychological distress and depression [12]. Women suffering from SARS-CoV-2 infection or pandemic-associated stress and anxiety were more likely to experience irregular menstruation, dysmenorrhea, amenorrhea, and other menstrual abnormalities compared to those who were less exposed [13]. Takmaz et al. [14] showed the association between increased prevalence of menstrual cycle irregularity and the COVID-19 pandemic-induced anxiety, perceived stress as well as depressive symptoms among healthcare providers. Maher et al. found increased levels of psychological distress and poor sleep are associated with menstrual cycle disruption [15]. Moreover, COVID-19 vaccination was associated with an immediate and temporary menstrual change, including irregular menstruation, abnormally heavy or prolonged bleeding, increased premenstrual symptoms and worse dysmenorrhea [8, 16].

SARS-CoV-2 infection could affect the menstrual cycle. Cycle length, period, blood volume, dysmenorrhea along with bleeding between menstruations all show different changes due to SARS-CoV-2 infection [17, 18]. Women have mainly reported decreased menstrual blood volume and a prolonged cycle [19]. Ding et al. found the menstrual change was related to systemic complications, mainly diabetes, liver disease and malignant tumors [20]. People that reported changes in their menstrual cycle after SARS-CoV-2 infection reported more COVID-19 symptoms than those who did not [18]. Our study also found that those who experienced chest pain and dyspnea during infection were more likely to experience an irregular menstrual cycle. But age, comorbidities, and the severity of COVID-19 did not play a role in menstrual cycle changes. Furthermore, the timing of being infected during the menstrual cycle did not affect the cycle length.

The mechanism by which COVID-19 causes changes in menstruation remains unclear. It may be mediated by both ovarian hormones (affecting cycle length) and endometrial repair (affecting menstrual blood volume). Ding et al. [20] provides the initial clinical evidence showing that female COVID-19 patients probably have an ovarian injury of poor ovarian reserve and reproductive endocrine disorder with decreased AMH and aberrant sex hormone levels, especially high T and PRL. The results inferred a potentially diminished ovarian reserve and reduced reproductive potential in a short time. Direct virus attack, excessive immune, inflammatory response and dysfunction of HPO axis may all contribute to the abnormal ovary function under COVID-19, leading to ovarian injury at last, including declined ovarian reserve and reproductive endocrine disorder in women with COVID-19. While other studies suggested that the SARS-CoV-2 virus does not impact sex hormone concentrations and ovarian reserve [21, 22], Miguel-Gómez et al. [23] found COVID-19 altered endometrial gene expression in 75% of the women, including up-regulation in pathways of immune responses to viruses and cytokine inflammation.

COVID-19, as an illness, increasing morbidity rate by not only the infection itself but also the immune response to the virus. Viewing COVID-19 as an inflammatory process, it has also given rise to the hypotheses regarding the development of long-COVID and other post-COVID associated morbidities. Studies have addressed the hypothesis that menstrual changes after SARS-CoV-2 infection are associated with activation of the immune response [24–26]. Two biologically mechanisms that immune stimulation might cause menstrual changes have been proposed: Innate immune responses could transiently interfere with the hormones that drive the menstrual cycle [27], or they could affect macrophages and natural killer cells in the lining of the uterus, which control the breakdown and regeneration of endometrium through the cycle [28].

Our study has limitations. First of all, it is a retrospective study including only Chinese population and we did not collect information on the mental health of the participants. Secondly, data of menstrual cycle characteristics, SARS-CoV-2 infection and vaccination were self-reported, and premenopausal women with ovulational disorders were not excluded from the study. Thirdly, online surveys select internet users and are more likely to be completed and shared among people affected by the studied condition. It also presents limitations to population generalizability that participants of our study are mainly healthcare workers. Last but not least, Mid-December 2022 was the peak of the pandemic in China, so the time frame is short. We did not inspect the long-term impact of SARS-CoV-2 infection.

## Conclusions

Our study found that COVID-19 was associated with AUB. Women infected with SARS-COV-2 were more likely to have irregular menstrual cycles than uninfected women. COVID-19 vaccination is a protective factor in the long term, and those with chest pain and dyspnea are more likely to develop AUB. Our findings suggested the need to get COVID-19 vaccination and monitor menstrual cycle health in women with SARS-CoV-2 infection. Further researches are needed to understand the underlying mechanisms for these associations.

## Data Availability

The data that support the findings of this study are available from the corresponding author upon reasonable request, subject to institutional and ethical board approvals.

## Abbreviations

AUB: abnormal uterine bleeding
COVID-19: coronavirus disease 2019
HPO: hypothalamic-pituitary-ovarian
SARS-COV-2: severe acute respiratory syndrome coronavirus-2
